# Disarming Cellular Alarm Systems—Manipulation of Stress-Induced NKG2D Ligands by Human Herpesviruses

**DOI:** 10.3389/fimmu.2017.00390

**Published:** 2017-04-11

**Authors:** Dominik Schmiedel, Ofer Mandelboim

**Affiliations:** ^1^Faculty of Medicine, The Lautenberg Center for General and Tumor Immunology, The BioMedical Research Institute Israel-Canada, The Hebrew University Hadassah Medical School, Jerusalem, Israel

**Keywords:** NKG2D ligands, stress-induced ligands, NKG2D, herpesvirus, host-pathogen interaction, immune evasion, coevolution

## Abstract

The coevolution of viruses and their hosts led to the repeated emergence of cellular alert signals and viral strategies to counteract them. The herpesvirus family of viruses displays the most sophisticated repertoire of immune escape mechanisms enabling infected cells to evade immune recognition and thereby maintain infection. The herpesvirus family consists of nine viruses that are capable of infecting humans: herpes simplex virus 1 and 2 (HSV-1, HSV-2), varicella zoster virus (VZV), Epstein–Barr virus (EBV), human cytomegalovirus (HCMV), roseoloviruses (HHV-6A, HHV-6B, and HHV-7), and Kaposi’s-sarcoma-associated herpesvirus (KSHV). Most of these viruses are highly prevalent and infect a vast majority of the human population worldwide. Notably, research over the past 15 years has revealed that cellular ligands for the activating receptor natural-killer group 2, member D (NKG2D)—which is primarily expressed on natural killer (NK) cells—are common targets suppressed during viral infection, i.e., their surface expression is reduced in virtually all lytic herpesvirus infections by diverse mechanisms. Here, we review the viral mechanisms by which all herpesviruses known to date to downmodulate the expression of the NKG2D ligands. Also, in light of recent findings, we speculate about the importance of the emergence of eight different NKG2D ligands in humans and further allelic diversification during host and virus coevolution.

## Herpesviruses—Constant Companions During Human Life and Evolution

Herpesviruses have accompanied humankind since the dawn of evolution. Herpesvirus infections date back at least 6 million years, even before evolutionary split between hominids and chimpanzees (1). From that time on, viral strategies to ensure survival and dissemination coevolved together with the immune system that continuously developed new measures to clear viral infections.

To date, nine different herpesviruses capable of infecting humans have been identified: HSV-1, HSV-2, varicella zoster virus (VZV), Epstein–Barr virus (EBV), human cytomegalovirus (HCMV), HHV-6A, HHV-6B, HHV-7, and Kaposi’s-sarcoma-associated herpesvirus (KSHV) (2).

Despite their different life cycle and growth properties, cellular tropisms and although they cause different diseases, all herpesviruses share common features. They are enveloped and contain a linear, double-stranded DNA genome, ranging from 125 kb (for VZV) to 235 kb (for HCMV) (2). Furthermore, all have the ability not only to infect lytically but also to establish life-long infection in their host, a status called latency, which is a dormant infection lacking pathology and viral replication (2, 3).

Most herpesviruses are widely spread in human populations. Serological tests reveal that HSV-1, VZV, EBV, HHV-6, and HHV-7 have the highest prevalence of the herpesvirus family and infect about 90% of the adult population (4–10). Notably, the prevalence of herpesviruses varies geographically and is influenced by socioeconomic status (2, 11, 12). HCMV prevalence can therefore vary between 50 and 100% dependent on the population studied (13). Some herpesviruses reactivate symptomatically and frequently in healthy individuals for as yet unknown reasons, while others only cause symptomatic reactivation in immunodeficient patients (3). However, research over the past few years revealed that all herpesviruses use common strategies during primary infection, reactivation, and sometimes even during latency, in order to evade the immune surveillance during the different phases of herpesvirus life cycle. The interactions between adaptive immunity and herpesviruses are described elsewhere (14–17). In this review, we will focus on the interaction of herpesviruses and natural-killer group 2, member D (NKG2D)-expressing immune cells. The human-activating receptor NKG2D is expressed on all natural killer (NK) cells as well as on most T cells including γδ T cells and NK T cells (18). Its importance was shown for tumor surveillance (19) and inflammatory diseases (20). The significance of NK cells in herpesvirus immune surveillance becomes clear by looking at NK cell-deficient individuals who suffer from recurrent, severe, potentially life-threatening herpesvirus infections (21, 22).

## Genetics of NKG2D Ligands

In the course of human evolution, eight different, functional ligands for the NKG2D receptor emerged: MHC class I polypeptide-related sequence A and B (MICA and MICB, respectively) and the unique long 16 binding protein 1–6 (ULBP1–6) (23). Also known as “stress-induced ligands,” they are barely found on healthy cells in order to avoid auto-reactivity toward normal tissues. These ligands, however, are upregulated and expressed on the cell surface following various stresses including genotoxic stress, oncogene activation or hypoxia that are commonly seen in tumorigenesis, or following viral infection (24, 25).

All NKG2D ligands belong to the MHC class I-like protein family. ULBP family members have an α1/α2 domain structure, whereas the MIC proteins possess an α1/α2/α3 domain structure (26). Interestingly, classical MHC class I proteins serve mainly as inhibitory ligands for NK cells, whereas the NKG2D ligands activate NK cells (27, 28).

**Figure 1:**
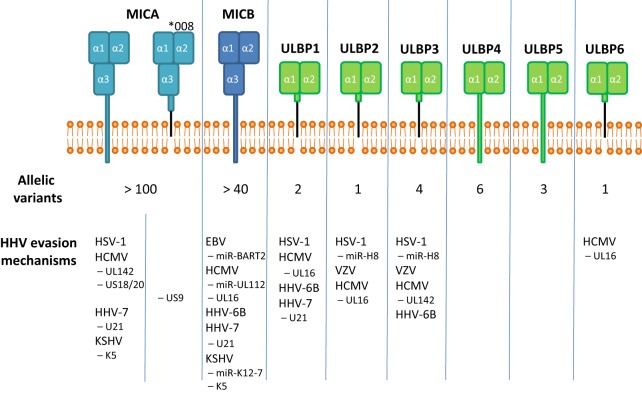
**The human genome encodes for eight functional stress-induced ligands, subdivided in the MIC and ULBP family**. MICA, MICB, ULBP4, and ULBP5 contain a transmembrane domain, whereas ULBP1, 2, and 3 and one particular allele of MICA, MICA*008, are GPI-anchored. Interestingly, MICA and MICB, genes having a high evolutionary plasticity as reflected by the number of allelic variants, seem to be targeted more frequently by viral immune evasion mechanisms.

Up until now, 16 different allelic variants were identified for the 6 members of the ULBP family (29). More than 100 different MICA alleles and more than 40 MICB alleles were identified to date; a finding that demonstrates the striking superior evolutionary plasticity of the MIC family [http://hla.alleles.org/alleles/classo.html; (30)] (Figure 1). The reason behind this enormous diversity of the MIC family is still unknown. MIC genes lack hypervariable regions; point mutations and genetic shuffles occur over all three domains (31). Comparing amino acid sequence homology, MICA and MICB are very similar (about 85% identity), whereas the similarity to ULBP family proteins is comparatively low (only about 20–25% identity between MIC and ULBP proteins). ULBP family members shares about 60% amino acid sequence identity with each other (32–34). Interestingly, MICA, MICB, ULBP4, and ULBP5 contain a transmembrane domain and a cytoplasmic tail, whereas ULBP1, ULBP2, ULBP3, ULBP6, and one particular allelic variant of MICA (allele *008) are glycosylphosphatidylinositol (GPI) anchored (30).

Only recently, post-transcriptional cellular mechanisms that control stress-induced ligand expression by RNA-binding proteins (35–37) and microRNAs (miRNAs) (38, 39) began to be unraveled; however, the regulatory circuits and expression patterns in normal cells remain incompletely understood. By contrast, much information was gathered about the suppression of NKG2D ligands during herpesvirus infection, emphasizing the importance of the receptor NKG2D for anti-viral immunity.

## All Herpesviruses Suppress Expression of Stress-Induced Ligands During Infection

### HSV-1 and -2—HHV-1 and -2

Herpes simplex virus 1 and 2 can cause orofacial and genital infections in elsewise healthy individuals with a competent immune system (40). Reactivation is believed to be triggered by stress, sunlight, fever, or skin traumas, e.g., caused by surgery (40, 41).

The effects of HSV-1 infection on the expression of NKG2D ligands were first studied by Schepis et al. [(42); Figure 1; Table 1]. Both ULBP2 and MICA surface expression levels were found to be decreased following infection with HSV-1 strain F. Concurrent with a loss of surface expression, MICA messenger RNA (mRNA) levels were decreased. Since MICA downregulation was abrogated by inhibiting the viral DNA polymerase, the authors concluded that a late viral gene is responsible for the reduction of MICA expression. However, in this study, none of the cell lines tested expressed other ligands besides MICA and ULBP2. Another study, performed by Campbell et al., confirmed the decrease in MICA and ULBP2, but could additionally show a downregulation of ULBP1 and ULBP3 (43). Interestingly, MICA, ULBP2, and ULBP3 were shown to be reduced at the overall protein level, whereas ULBP1 was retained intracellularly, proving that different mechanisms act on these ligands (43).

**Table 1:** **Overview of known suppression mechanisms for NKG2D ligands by HHVs**.

	Viral effector	Ligand	Mechanism	Reference
HSV-1	?	MICA	?	(42, 43)
	?	ULBP1	Intracellular retention	(43)
	miR-H8	ULBP2	Interferes with protein maturation	(42–44)
	miR-H8	ULBP3	Interferes with protein maturation	(43, 44)
HSV-2	?	?	?	?
Varicella zoster virus	?	ULBP2	Intracellular retention	(43)
	?	ULBP3	Intracellular retention	
Epstein–Barr virus	miR-BART2-5p	MICB	Translational repression	(53)
Human cytomegalovirus	miR-UL112	MICB	Translation repression	(56)
	UL16	MICB	Intracellular retention	(60)
		ULBP1	Intracellular retention	(32, 57)
		ULBP2	Intracellular retention	(32, 57)
		ULBP6	Intracellular retention	(61)
	UL142	MICA	Intracellular retention	(62)
		ULBP3	Intracellular retention	(63)
	US18/US20	MICA	Lysosomal degradation	(64)
	US9	MICA*008	Proteasomal degradation	(68)
HHV-6A	?	?	?	?
HHV-6B	?	MICB	Proteasomal degradation	(71)
	?	ULBP1	Proteasomal degradation	
	?	ULBP3	Proteasomal degradation	
HHV-7	U21	MICA	?	(72)
		MICB	?	
		ULBP1	Lysosomal degradation	
Kaposi’s-sarcoma-associated herpesvirus	K5	MICA	Ubiquitinylation/intracellular retention	(75)
		MICB	Ubiquitinylation/intracellular retention	(75)
	miR-K12-7	MICB	Translational repression	(53)

*?, no published data available*.

In a subsequent study, Enk et al. added some mechanistic detail about the regulation of ULBP2 and ULBP3 (44). They reported that the viral miRNA miR-H8 interferes with the generation of GPI-anchored proteins by targeting PIGT, a key protein in the GPI-anchoring process (45, 46). Consequently, both ULBP2 and ULBP3 levels were reduced in miR-H8 overexpressing cells. Interestingly, ULBP1 is also GPI anchored but not affected by this pathway, explaining the necessity for another mechanism of downregulation—intracellular retention. However, since both MICA (except the allele MICA*008) and MICB are transmembrane proteins and were not affected by the miR-H8 overexpression, the viral mechanism responsible for these downregulations are still unknown.

### VZV—HHV-3

Varicella zoster virus is the causative agent of varicella (chickenpox) in primary infection (47). In the elderly or immunosuppressed patients, VZV can reactivate and cause herpes zoster (shingles), which is characterized by painful skin lesions as well as neurological and ocular disorders (47, 48).

By infecting retinal epithelial cells with a clinical VZV strain, Campbell et al. revealed a down-modulation of the surface expression of the NKG2D ligands ULBP2 and ULBP3 [(43); Figure 1; Table 1]. By contrast, MICA surface expression increased during the course of VZV infection; ULBP1 and MICB were not expressed in the studied cells. Due to the overall reduction of surface expression of NKG2D ligands, reduced activation of NK cells in the presence of VZV-infected cells as compared to mock infected cells was observed. Interestingly, the total protein levels of ULBP2 and ULBP3 were not reduced in infected cells (43), indicating intracellular retention of these ligands by a yet unknown viral factor.

### EBV—HHV-4

Epstein–Barr virus is usually acquired asymptomatically in childhood (2, 49). Infection during adolescence can lead to infectious mononucleosis (in about 50% of primary infections), a weakening and sometimes painful but self-limiting disease associated with the occurrence of atypical lymphocytes in the blood stream (2, 49). Reactivation can occur in immunocompromised individuals and is, among others, linked not only to lympho-proliferative diseases such as Burkitt’s and Hodgkin’s lymphoma but also to nasopharyngeal carcinoma (50, 51).

A sensitization of EBV-infected cells switching from latent to lytic infection to NK cell killing was reported by Pappworth et al. (52). They showed the induction of ULBP1 following this switch in a Burkitt’s lymphoma-derived cell line, whereas all other NKG2D ligands were absent from the cell surface. Later on, an overexpression study performed by Nachmani et al. revealed that the latency-associated viral miRNA miR-BART2-5p is capable of binding MICB mRNA and suppressing its translation [(53); Figure 1; Table 1]. Interestingly, they showed that the binding site in the MICA mRNA sequence was mutated in such a way that prevented the miRNA from suppressing MICA as well.

Remarkably, to the best of our knowledge, there are no immune evasion mechanisms regarding NKG2D ligands during lytic EBV infection described to date. This phenomenon might be explained by a study published by Song et al. (54). They showed that EBV-transformed B cells produce and release the tryptophan-derived metabolite l-kynurenine that downmodulates NKG2D receptor expression on by-stander NK cells. Therefore, the suppression of NKG2D ligands on infected cells might be of little importance if the effector cells themselves are effectively disarmed.

### HCMV—HHV-5

While being a harmless pathogen for immunocompetent individuals, HCMV constitutes a major risk for the elderly, patients after organ transplantation and AIDS patients (55). Additionally, primary infection in pregnant women can cause miscarriage, stillbirth, or developmental retardation of the child (55). HCMV possesses the largest genome of all HHVs of about 235 kb (2). Therefore, it might not be surprising that HCMV has the greatest number of viral mechanisms dedicated to the immune evasion by manipulating NKG2D ligands described to date.

The first viral miRNA identified to target immune molecules in general and NKG2D ligands in particular was miR-UL112, discovered by Stern-Ginossar et al. [(56); Figure 1; Table 1]. By binding to the 3′-UTR of the MICB mRNA, it represses translation, and surface levels are rapidly reduced, leading to decreased NK cell activation. UL16 was the first HCMV viral protein found to bind and retain ULBP1, ULBP2, ULBP6, and MICB intracellularly (“ULBPs” were named for being UL16-binding proteins) (32, 57–61). Later, UL142 was shown to sequester both MICA and ULBP3 intracellularly, they colocalized with markers of the *cis*-Golgi apparatus inside infected cells (62, 63).

Fielding et al. showed that the viral proteins US18 and US20 are capable of both independently and synergistically downregulating MICA expression by targeting it for lysosomal degradation (64).

Notably, the GPI-anchored allele MICA*008 was not found to be targeted by the abovementioned viral mechanisms and was therefore considered as HCMV-resistant escape variant. Since the MICA*008 allele is a highly prevalent in human populations worldwide, the hypothesis was formed that its prevalence is the result of viral selective pressure (65–67). However, Seidel et al. showed that this supposed escape variant is specifically targeted by the HCMV protein US9 during its maturation process, prior to its egress from the ER, instead forcing MICA*008 to proteasomal degradation (68).

### Roseoloviruses—HHV-6A, HHV-6B, and HHV-7

HHV-6A, -6B, and -7 have long been neglected in research. Only in the past years have these viruses gained attention since it became obvious that they not only cause a common children’s disease (roseola infantum) but might also be involved in severe illnesses, especially in immunoincompetent individuals like neuroinflammatory diseases (HHV-6A), transplant rejection, myocarditis (HHV-6B), or encephalitis (HHV-6A, -6B, and HHV-7) (69, 70). For this reason, immunomodulatory features of these viruses were studied only relatively recently.

We showed that HHV-6B strain Z29 is capable of suppressing the surface expression of the NKG2D ligands ULBP1, ULBP3, and MICB, but not MICA or ULBP2 [(71); Figure 1; Table 1]. This was true both in primary T cells and in T cell lines. As a cellular response to the viral infection, mRNA levels of all stress-induced ligands rise following infection; however, the virus suppresses the three abovementioned ligands on protein level and degrades them rapidly in a proteasome-dependent pathway shortly after the start of infection. Also, we showed that the degradation of the three ligands is mediated by at least two different viral proteins.

As for HHV-7, Schneider et al. showed that U21, which was previously shown to target HLA class I for lysosomal degradation, also causes lysosomal degradation of ULBP1 resulting in a mild downregulation. Additionally, they observed a major downregulation of MICA and MICB (72). These findings were established using the overexpression of the viral protein U21. However, the exact mechanism for MIC proteins degradation remained unclear. Probably, U21 interferes with proper protein glycosylation rendering the MIC proteins unstable and causing them to be targeted for cellular degradation. Due to the mild loss of ULBP1, this degradation was proposed to be the result of a “side-effect” of U21-mediated HLA class I degradation, since these related proteins were targeted to lysosomal degradation with higher affinity and to a greater extent.

However, since the study was limited to overexpression of a single gene and no studies were conducted using an actual infection model, it is possible that additional stress-induced ligands are affected by HHV-7 or that additional mechanisms targeting the same ligands exist.

### KSHV—HHV-8

Kaposi’s-sarcoma-associated herpesvirus is the human herpesvirus with the lowest seroprevalence in the Western world with only about 1–3% of individuals infected (73). Still, this virus is a significant cause of cancer, primarily in AIDS patients, whereas immunocompetent individuals do not experience KSHV reactivation (73, 74). In developing countries, seroprevalence is substantially higher (73).

During lytic infection, KSHV evades NK cell recognition by expressing the viral E3 ligase K5. Thomas et al. showed that K5 modifies lysine residues within the cytoplasmic tails of both MICA and MICB with ubiquitin. Consequently, these molecules are internalized from the cell membrane and intracellularly sequestered, but not degraded [(75); Figure 1; Table 1]. Notably, the fact that the MICA allele *008 as well as ULBP1, ULBP2, and ULBP3 are GPI anchored and therefore lack a cytoplasmic tail, render them resistant to K5-mediated ubiquitinylation. Additionally, Nachmani et al. reported that the viral miRNA miR-K12-7 specifically represses the translation of MICB by binding to the 3′-UTR of its mRNA (53). Interestingly, MICA mRNA was shown not to be targeted by miR-K12-7 since the 3′-UTR is significantly shorter than the MICB equivalent and does not contain the binding site (53).

## Eight Ligands, Further Allelic Diversification: Host–Pathogen Evolution at Full Speed

As emphasized above, herpesvirus family members developed numerous mechanisms to interfere with the expression of the stress-induced ligands. However, most of these studies still leave unanswered questions. More mechanisms and viral effectors are still waiting to be discovered. The viral protein repertoire is probably much larger than currently known; by using ribosome profiling of HCMV and KSHV, numerous new open reading frames (ORFs) have been identified (76, 77). The functions of many viral proteins and ORFs are yet unknown and we are just on the verge of understanding the importance of viral non-coding RNAs, including long non-coding RNAs (78, 79).

While the NKG2D receptor itself is conserved among species, its ligands are not. Interestingly, having eight functional ligands of two different families (MIC and ULBP) and various alleles, the human NKG2D ligand repertoire is more complex than that of other species. Mice possess even nine functional ligands (MULT1, Raet1α–ε, H60a–c) (80). However, their domain structure reveals them to be ULBP family homologs with low allelic diversity. Non-human primates were shown to have homologs of the MIC proteins (81, 82). Still, compared to humans with more than 100 allelic variants, even great apes seem to possess lower allelic variation (83).

Herpesviruses might be a major driving force for diversification of stress-induced ligands and further mutagenesis within alleles leading to allelic variations. None of the described viral mechanisms is capable of eliminating the expression of all stress-induced ligands, the evolutionary pressure rendered these ligands so diverse that no single viral protein or RNA is sufficient to regulate all of them.

As described earlier, viral miRNAs of HCMV, EBV, and KSHV target MICB mRNA at different sites of its 3′-UTR and suppress protein translation (53, 56). Despite the high degree of sequence homology in their 3′-UTRs, MICA is not targeted by any of these miRNAs. The binding sites for the viral miRNAs of HCMV (miR-UL112) and EBV (miR-BART2-5p) are modified by a single-nucleotide insertion, thus abolishing miRNA-induced translation repression. The sequence that is targeted by the KSHV encoded miR-K12-7 is completely absent due to a major deletion in the MICA 3′-UTR.

A similar mutagenesis apparently occurred in the MICA protein to escape UL16 binding. UL16 binds to an α-helical structure in the α2 domain of MICB. By substituting single amino acid residues in MICB with their MICA equivalents, Spreu et al. could show that a single substitution (at two different positions) is sufficient to abolish UL16 binding (84); hence, MICA is spared from UL16-mediated intracellular retention by virtue of very few mutations. Additionally, Klumkrathok et al. suggested that even different allelic variants of MICB are bound with different affinities by UL16 due to amino acid substitutions in the α2 domain (85).

A third piece of evidence for a herpesvirus-driven coevolution is the emergence of MICA*008, a highly prevalent, GPI-anchored MICA variant. MICA*008 is not targeted by UL142, by US18, or by US20. Only recently, it was discovered that the evolutionary relatively novel US9 is capable of targeting solely this distinct allele, but none of the full-length alleles containing a transmembrane domain (68).

These few examples illustrate well the human capability to adjust to viral immune evasion strategies. Accordingly, this strong selective, coevolutionary pressure necessitates modification of viral effector molecules targeting the immune surveillance system as well. By comparing different isolates from HCMV-infected individuals, Renzette et al. and Sijmons et al. indeed showed on a global level that genes involved in immune evasion within the HCMV genome are strongly diversified and contain high numbers of single-nucleotide polymorphisms (86, 87). Among others, one particular mutable gene was found to be UL142, which interacts with NKG2D ligands as pointed out before (87).

## Diversification: An Evolutionary Necessity?

It seems obvious to conclude that herpesviruses and ligands for NKG2D continuously shape each other during coevolution, whereas the NKG2D receptor itself remains conserved.

Particularly MICA took the lead in this race on the human side, its 3′-UTR became shortened and modified and numerous allelic variations emerged to withstand herpesvirus infection. The diversity of MICA alleles might thereby even create a “population level resistance” by making it difficult for newly emerging viral mechanisms to successfully target all MICA variants at once.

However, in contradiction to this theory and the supposed importance of stress–ligand evolution, several reports showed a wide distribution of a MICA–MICB null haplotype (also described as MICA-del–MICB-null), a phenotype that occurs mainly, but not exclusively, in East Asia (88–91), apparently with no major evolutionary disadvantage or clinical manifestations. In fact, there are several known MICA-null alleles also independent of this haplotype. If and how MICA and MICB functions are compensated in these individuals, e.g., by the redundancy of the other NKG2D ligands that are still present, has yet to be elucidated; however, this phenomenon teaches us that we are still far from a complete understanding of the complex families of NKG2D ligands.

## Author Contributions

DS outlined, wrote, referenced the manuscript, and prepared the figure and table. OM supervised and carefully edited the work.

## Conflict of Interest Statement

The authors declare that the research was conducted in the absence of any commercial or financial relationships that could be construed as a potential conflict of interest.
